# Combined effect of regulatory polymorphisms on transcription of *UGT1A1 *as a cause of Gilbert syndrome

**DOI:** 10.1186/1471-230X-10-57

**Published:** 2010-06-08

**Authors:** Katsuyuki Matsui, Yoshihiro Maruo, Hiroshi Sato, Yoshihiro Takeuchi

**Affiliations:** 1 Department of Pediatrics, Shiga University of Medical Science, Tsukinowa, Seta, Otsu, Shiga 520-2192, Japan; 2 Department of Bioscience, Shiga University of Medical Science, Tsukinowa, Seta, Otsu, Shiga 520-2192, Japan

## Abstract

**Background:**

Gilbert syndrome is caused by defects in bilirubin UDP-glucuronosyltransferase (UGT1A1). The most common variation believed to be involved is A(TA)7TAA. Although several polymorphisms have been found to link with A(TA)7TAA, the combined effect of regulatory polymorphisms in the development of Gilbert syndrome remains unclear.

**Methods:**

In an analysis of 15 patients and 60 normal subjects, we detected 14 polymorphisms and nine haplotypes in the regulatory region. We classified the 4-kbp regulatory region of the patients into: the TATA box including A(TA)7TAA; a phenobarbital responsive enhancer module including c.-3275T>G; and a region including other ten linked polymorphisms. The effect on transcription of these polymorphisms was studied.

**Results:**

All haplotypes with A(TA)7TAA had c.-3275T>G and additional polymorphisms. In an *in-vitro *expression study of the 4-kbp regulatory region, A(TA)7TAA alone did not significantly reduce transcription. In contrast, c.-3275T>G reduced transcription to 69% of that of wild type, and the linked polymorphisms reduced transcription to 88% of wild type. Transcription of the typical regulatory region of the patients was 56% of wild type. Co-expression of constitutive androstane receptor (CAR) increased the transcription of wild type by a factor of 4.3. Each polymorphism by itself did not reduce transcription to the level of the patients, however, even in the presence of CAR.

**Conclusions:**

These results imply that co-operation of A(TA)7TAA, c.-3275T>G and the linked polymorphisms is necessary in causing Gilbert syndrome.

## Background

The elimination pathway of bilirubin in humans is catalyzed exclusively by bilirubin UDP-glucuronosyltransferase (UGT1A1) [1]. Gilbert syndrome is a mild hereditary unconjugated hyperbilirubinemia without liver dysfunction or hemolytic anemia. It is caused by defects of UGT1A1 and is one of the most prevalent congenital metabolic disorders; Gilbert syndrome is found clinically in 3-10% of the population [2-5].

The UGT1A1 gene (*UGT1A1*) has the TATA box and a phenobarbital responsive enhancer module (gtPBREM) in its regulatory region [6,7]. The wild type of TATA box has six repeats of TA, and is conventionally written as A(TA)6TAA [6]. The gtPBREM is an important enhancer module of *UGT1A1*, comprising a region of 290-bp. It is located approximately 3.5 kbp upstream from the coding region and works in the presence of nuclear receptors, such as constitutive androstane receptor (CAR) [7].

Three variations are the most common polymorphisms in patients with Gilbert syndrome: the TA-insertion (c.-53TA[8]) in the TATA box, written as A(TA)7TAA; c.-3275T>G in gtPBREM; and c.211G>A (p.G71R) in exon 1 [8-11]. In previous reports, c.-3275T>G has been written as c.-3279T>G [12]. In the present report we adopted the nucleotide sequence of *UGT1A1 *revealed in GenBank Accession No. NG_002601, based on our gene analysis. A(TA)7TAA is found in all ethnic groups, and its frequency is 0.36-0.40 in Caucasians, 0.35-0.43 in Africans and 0.15 in Japanese [11-15]. Most Caucasians and Africans with Gilbert syndrome have homozygous A(TA)7TAA [11]. Consequently, A(TA)7TAA is believed to be the main cause of Gilbert syndrome.

UGT1A1 activity in patients with Gilbert syndrome was about 30% of normal, based on studies involving human liver samples [16-18]. In subsequent studies using human liver samples of homozygous A(TA)7TAA, UGT1A1 enzyme activity was 48% of wild type, and the expression level of UGT1A1 protein was 42-50% of wild type [18-20]. A(TA)7TAA reportedly has only one-third of the transcriptional activity of wild type, based on a gene expression study with the regulatory region from c.-546 to c.-4 [11]. No statistically significant difference in activity between A(TA)6TAA and A(TA)7TAA was detected in our own earlier study, however, using six different lengths of regulatory region varying from 111 to 3188-bp in length [21]. Also, other groups reported that transcriptional activity was not so drastically reduced by A(TA)7TAA (to 70-86% of wild type, not by one-third) [14,22].

The frequency of c.-3275T>G in gtPBREM is 0.26 in Japanese, 0.47 in Caucasians and 0.85 in African-Americans [12,23]. We found that all patients with Gilbert syndrome having homozygous A(TA)7TAA were also homozygous for c.-3275T>G, and we suggested that a combined effect of these polymorphisms on transcription is essential to the syndrome [24]. Furthermore, the c.-3152G>A and c.-364C>T polymorphisms, as well as c.-3275T>G, were recently found to link with A(TA)7TAA [12,23], and numerous single nucleotide polymorphisms (SNPs) of *UGT1A1 *were also reported on GeneSNPs [25]. To determine the role of the regulatory polymorphisms in Gilbert syndrome, we first analyzed haplotypes of the patients and normal subjects. Based on the results, the transcriptional activity of a continuous 4-kbp regulatory region was studied in the presence and absence of CAR.

## Methods

### Sequencing of the *UGT1A1 *transcriptional regulatory region

The upstream region of *UGT1A1 *were analyzed in a random population of 50 Japanese subjects and in four Japanese with Gilbert syndrome having homozygous A(TA)7TAA, as well as in ten healthy normal Caucasians and 11 unrelated Caucasians with Gilbert syndrome; all gave informed consent. The study was approved by the ethics committee of Shiga University of Medical Science. We amplified the regulatory region as four separate DNA fragments, using the polymerase chain reaction (PCR). Prime Star HS (TaKaRa BIO INC., Shiga, Japan) was used for PCR of fragment 1 (c.-3559 to c.-2614). Ex Taq (TaKaRa BIO INC) was used for PCR of fragment 2 (c.-2670 to c.-883), fragment 3 (c.-950 to c.-314) and fragment 4 (c.-446 to c.879 + 78). The D buffer of a PCR Optimizer Kit (Invitrogen Corporation, Carlsbad, CA) was used instead of the attached buffer of Ex Taq in the PCR of fragment 3. PCR products were sequenced directly using an ABI PRISM^® ^Big Dye^® ^Terminator v1.1 Cycle Sequencing Kit (Applied Biosystems, Foster City, CA). Tables 1-A and 1-D show all primers and conditions used in this study.

**Table 1:** Primers for amplification and sequencing of *UGT1A*

Primer name	Sequence (5' to 3')	Condition
Amplification of *UGT1A1 *transcriptional regulatory region for direct sequence		

Amp1-F	CACCTCCTCCTTATTCTCTT^a^	fragment 1
Amp1-R	CCTTGAATTTCCAAAATCCCAGA	3 min at 96°C, 35 cycles (20 sec at 96°C, 15 sec at 57°C, 90 sec at 72°C), 5 min at 72°C

Amp2-F	GATACAAGGCAGAACAGAAC	fragment 2
Amp2-R	AGGTCACACGGTTACTCTGA	2 min at 94°C, 35 cycles (20 sec at 94°C, 30 sec at 61°C, 70 sec at 72°C), 5 min at 72°C

Amp3-F	TGAGCGCTGAAAATCTCAAC	fragment 3
Amp3-R	AGAGAGGAAGAAGGACGACT	3 min at 94°C, 32 cycles (30 sec at 94°C, 45 sec at 59°C, 60 sec at 72°C), 10 min at 72°C

Amp4-F	ACAGGTTTCCATGGCGAAAG	fragment 4
Amp4-R	GCTTGCTCAGCATATATCTCTGGG^b^	2 min at 94°C, 35 cycles (20 sec at 94°C, 30 sec at 61°C, 60 sec at 72°C), 5 min at 72°C

Amplification of proximal and distal regulatory regions from genome DNA for cloning		

Proximal-F1	GACTGCCATCCAGTAGGGCTCACACGTT	
Proximal-R1	CGCCTTTGCTCCTGCCAGAGGTTCG	2 min at 94°C, 30 cycles (20 sec at 94°C, 5 min at 68°C), 10 min at 68°C

Distal-F1	GAGATCTGAGTTCTCTTCACCTCCTCCT	
Distal-R1	GCAGAGCTTCCAAGCTTTTTGAGGCTG	2 min at 94°C, 30 cycles (20 sec at 94°C, 30 sec at 63°C, 2 min at 72°C), 5 min at 72°C

Addition of restriction enzyme site of cloned transcriptional regulatory regions by PCR (underline: *Sac I *site on Proximal-F2 and Distal-F2; *Xho I *site on Proximal-R2)		

Proximal-F2	GAGAGCTCCCTCAGCCCCTAGAGCACCATC	
Proximal-R2	CTCTCGAGGCGCCTTTGCTCCTGCCAGAGG	2 min at 94°C, 8 cycles (20 sec at 94°C, 30 sec at 60°C, 90 sec at 72°C), 5 min at 72°C

Distal-F2	GAGAGCTCGAAGGGATTAGTTTAGGACAACCCTCCTTC	
Distal-R1	GCAGAGCTTCCAAGCTTTTTGAGGCTG	2 min at 94°C, 8 cycles (20 sec at 94°C, 30 sec at 60°C, 90 sec at 72°C), 5 min at 72°C

Sequencing primers for transcriptional regulatory region of *UGT1A1*		

Seq1-1	TATTCTCTTTTTGACACTGG	
Seq1-2	GACCAAGGTTCCAGAAGTGGTGGTGA	
Seq1-3	CAATTACAGGGGATGGTGCTCTAG	
Seq1-4	CTTCCAATTCTGGCTGCACA	5 min at 96°C, 25 cycles (10 sec at 96°C, 5 sec at 50°C, 4 min at 60°C) ^c^
Seq1-5	GACGAAGGAATGAAACACAT	
Amp4-F	ACAGGTTTCCATGGCGAAAG	

Sequencing primers for cloned transcriptional regulatory regions		

Seq2-1	TCTGCTGTTGGCTGAATCTG	
Seq2-2	TATACACACGGCCTGCAAGT	
Seq2-3	CAGAATGGCTAGAGGGTAAG	
Seq2-4	ACAGAAACATGTCCAGAGCACTT	
Seq2-5	TGTCTGATGGTGGCCTACTA	
Seq2-6	TTGCTGCCCTGCTGTGTG	5 min at 96°C, 25 cycles (10 sec at 96°C, 5 sec at 50°C, 4 min at 60°C) ^c^
Seq2-7	CATCCAGGTACACAGCAGAA	
Seq2-8	TCATTCCACTGGCCCAAGAT	
Seq2-9	GGTTCCCAATCAGGTCCATT	
Seq2-10	TCACATGCGCTCCAGTGAAT	
Seq1-2, Seq1-4, Amp4-F		

^a^Sugatani et al. [10]^b^Maruo et al. [15]^c^Yamada et al. [35]

### Construction of expression vectors

To study the effect of the TATA box, gtPBREM, and the other polymorphisms in the regulatory region, we constructed luciferase expression vectors including the regulatory regions with differing combinations of these polymorphisms. We also constructed a CAR expression vector, to investigate the effect of CAR on transcription of the regulatory region with gtPBREM.

#### Cloning of the regulatory region in two separate DNA fragments

Two types of genomic DNA were chosen to construct expression vectors. One is a Gilbert-syndrome type from a Japanese patient with Gilbert syndrome having homozygous A(TA)7TAA. The other is wild type from a normal subject having identical sequence, reported in GenBank Accession No. NG_002601. The distal region (c.-4076 to c.-1945) and proximal region (c.-2483 to c.247) were amplified with PCR, using TaKaRa LA Taq^® ^Hot Start Version (TaKaRa BIO INC) (Table 1-B). The PCR products were cloned into a pCR^®^-XL-TOPO^® ^vector with a TOPO XL PCR cloning kit (Invitrogen Corporation), and the entire regions involved were checked by sequencing so as to eliminate any mistakes arising in the PCR amplification (Table 1-E).

#### Construction of expression vectors

**Figure 1:**
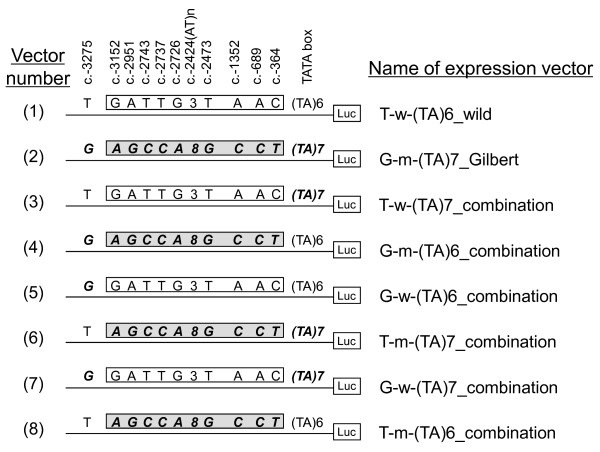
**List of vectors constructed**. *UGT1A1 *regulatory regions (c.-4076 to c.-1, Nos. 1 - 8), with differing combinations of polymorphic variations, were inserted into the luciferase reporter plasmid PGV-B2. The wild-type vector is No.1, and No.2 is a typical vector with regulatory regions showing Gilbert syndrome. The open box is a wild-type region between gtPBREM and the TATA box, including ten linked polymorphisms: c.-3152G>A, c.-2951A>G, c.-2743T>C, c.-2737T>C, c.-2726G>A, c.-2724AT[8], c.-2473T>G, c.-1352A>C, c.-689A>C and c.-364C>T. The gray box is a mutant-type region having mutant-type sequence corresponding to the wild-type region. Luc is luciferase cDNA. The name of the expression vector corresponds to the polymorphisms included and the type of vector. In the first part of the name, T and G respectively denote c.-3275T and c.-3275G. In the second part of the name, w and m refer to wild type and mutant-type region between gtPBREM and the TATA box. In the third part, (TA)6 and (TA)7 denote A(TA)6TAA and A(TA)7TAA. In the fourth part, wild, Gilbert and combination indicate that each vector has wild-type sequence, or typical mutant-type sequence in subjects suffering from Gilbert syndrome, or an artificial sequence constructed for this study.

Restriction sites for ligation into an expression vector were introduced into the proximal and distal regulatory regions by PCR of the cloned regulatory region, using PCR primers with the restriction sites (Table 1-C). Each PCR product (proximal; c.-2323 to c.-1: distal; c.-4076 to c.-1945) was inserted into the PCR^®^-XL-TOPO^® ^vector. PGV-B2 expression vectors (TOYO B-Net CO., LTD, Tokyo, Japan) having wild-type or Gilbert-syndrome-type continuous 4-kbp regulatory region (c.-4076 to c.-1) were constructed by restriction and ligation using a Ligation-Convenience Kit (NIPPON GENE CO., LTD., Tokyo, Japan), as listed in Figure 1: Vectors No.1 and 2 are wild and Gilbert-syndrome types, respectively.

We found that the typical region between gtPBREM and A(TA)7TAA of the patient included ten linked polymorphisms, c.-3152G>A, c.-2951A>G, c.-2743T>C, c.-2737T>C, c.-2726G>A, c.-2724AT[8], c.-2473T>G, c.-1352A>C, c.-689A>C and c.-364C>T (Table 2). The effect of the region on the transcriptional activity was determined as a unit. We classified the regulatory region of *UGT1A1 *with the detected polymorphisms into the following three regions: the TATA box including A(TA)7TAA, gtPBREM including c.-3275T>G, and the remaining region with the ten linked polymorphisms, according to their locations in the regulatory sequence and previous studies. To study the effects of the three regions on transcription, either solely or in combination, vectors with different combinations of polymorphisms were constructed by restriction and ligation, as listed in Figure 1. Vectors Nos. 3-8 are combination types.

**Table 2:** Haplotypes in the *UGT1A1 *transcriptional regulatory region

type	Number of allele				gtPBREM	Polymorphisms in the region between gtPBREM and TATA box												TATA box
				
	**JRS**^**a**^ **(100) **^**e**^	**JG7**^**b**^ **(8) **^**e**^	**NC**^**c**^ **(20) **^**e**^	**CG**^**d**^ **(22)**^**e**^	-3275	-3152	-2951	-2743	-2737	2726	-2724AT[n]	-2473	-1352	-1125	-997	-689	-364	A(TA)nTAA
					Haplotypes with A(TA)6TAA													
	
I	68	0	11	0	T	G	A	T	T	G	3	T	A	C	G	A	C	6
II	19	0	4	0	**G**	G	A	T	T	**A**	**>8**	**G**	**C**	C	**A**	**C**	C	6
III	0	0	1	0	T	G	A	T	T	G	3	**G**	**C**	C	**A**	**C**	C	6
IV	2	0	0	0	T	G	A	T	T	G	3	T	**C**	C	**A**	A	C	6
	
					Haplotypes with A(TA)7TAA													
	
V	10	8	0	0	**G**	A*	G*	C*	C*	A*	8*	G*	C*	C	G	C*	T*	**7**
VI	1	0	3	12	**G**	**A**	**G**	**C**	**C**	**A**	**>8**	**G**	**C**	C	G	**C**	**T**	**7**
VII	0	0	1	5	**G**	G	**G**	**C**	T	**A**	**>8**	**G**	**C**	C	G	**C**	**T**	**7**
VIII	0	0	0	4	**G**	G	**G**	**C**	**C**	**A**	**>8**	**G**	**C**	**T**	G	**C**	**T**	**7**
IX	0	0	0	1	**G**	**A**	**G**	**C**	**C**	**A**	**>8**	**G**	**C**	**T**	G	**C**	**T**	**7**

Boldfaces indicate variations.

^a^JRS, Japanese random subjects; ^b^JG7, Japanese patients with Gilbert syndrome having homozygous A(TA)7TAA; ^c^NC, normal Caucasians; ^d^CG, Caucasian patients with Gilbert syndrome; ^e^Numbers in parentheses indicate the number of total alleles in each group

*Ten linked polymorphisms between gtPBREM and TATA box

#### Construction of CAR expression vector

CAR-cDNA was isolated from a human liver cDNA library (TaKaRa BIO INC) by PCR, as described by Auerbach et al [26]. The PCR product was cloned into pCR2.1 vector (Invitrogen Corporation). CAR-cDNA was cut out by *EcoR *I and *EcoR *V, and ligated into the pCR3.1 vector (Invitrogen Corporation).

### Transfection and luciferase assay

HepG2 cells from the Riken Cell Bank (Tsukuba, Japan) were cultured in the minimal essential medium, which includes Earle's salts, non-essential amino acids but not - L-Glutamine (MEM, Invitrogen Corporation), supplemented with 10% fetal bovine serum (Invitrogen Corporation), 50 U of penicillin/ml and 50 μg of streptomycin/ml. The cells were transferred to a 24-well culture plate (7 × 10^4 ^cells/well) at 25 hours before transfection. For the transfection, 250 μl of MEM, 3.0 μl of GenePORTER^® ^Transfection Reagent (GeneLantis, San Diego, CA) and plasmid vectors were poured onto the cells in each well. The composition of the plasmid vectors for transfection was: luciferase reporter plasmid PGV-B2 with a transcriptional regulatory region (400 ng), the CAR-pCR3.1 expression plasmid or mock plasmid (200 ng), and the Renilla luciferase expression plasmid pRL-SV40 (6 ng) for normalization. After 3 hours, 250 μl of MEM containing 20% fetal bovine serum was added to each well. After 28 hours the transfected cells in each well were lysed, and the dual luciferase activities were immediately measured with a PicaGene Dual SeaPansy Luminescence Kit (TOYO B-Net CO., LTD).

### Data analysis

Haplotype analysis was carried out using Haploview 3.32 software [27]. Changes in transcriptional activity due to the polymorphisms were analyzed by multiple regression analysis (see Additional file 1, Table S1), using SPSS16.0.1J software (SPSS Japan Inc., Tokyo, Japan). For dummy coding, we defined the wild-type and mutant-type polymorphisms as 0 and 1, respectively.

## Results

### Sequencing and haplotype analysis in the regulatory region of *UGT1A1*

The upstream region of *UGT1A1 *was analyzed in patients with Gilbert syndrome and in normal Japanese and Caucasian subjects. The regulatory region of wild type was consistent with NG_002601, which is stated to be the sequence of *UGT1A1 *by the US National Center for Biotechnology Information (Table 2, type I). Fourteen polymorphic variations with distinct combinations were identified (Table 2). A(TA)7TAA always linked with c.-3275T>G, c.-2951A>G, c.-2743T>C, c.-2726G>A, c.-2473T>G, c.-1352A>C, c.-689A>C and c.-364C>T. In addition, the alleles with A(TA)7TAA had variants of the AT-repeat number from c.-2724. The AT-repeat number at c.-2724 of the wild type was three; we denote this as c.-2724AT [3]. The variant of Japanese patients having homozygous A(TA)7TAA was eight AT-repeats: c.-2724AT[8]. The repeat number was greater than eight in Caucasian patients, but could not be determined exactly because of the difficulty involved in reliable amplification with PCR. These variants are described here as c.-2724AT[>8]. Also, A(TA)7TAA was linked mostly with c.-3152G>A (c.-3156G>A in a previous report (10)) and c.-2737T>C. From the analysis, nine different haplotypes were identified, with distinct combinations of the 14 polymorphic variations (Table 2) and 14 kinds of diplotype (see Additional file 2, Table S2).

### Effect of regulatory polymorphisms on transcription

Based on haplotype analysis of Gilbert patients in Japanese and Caucasians, we selected the most typical regulatory regions of wild and Gilbert-syndrome type for *in-vitro *expression studies: in Table 2, type I and type V respectively. The regulatory region for Gilbert syndrome was cloned from a Japanese patient because of the uncertain number of AT-repeat in Caucasians, since all homozygotes with A(TA)7TAA were Gilbert syndrome irrespective of c.-2724AT[8] or c.-2724AT[>8]. To simplify the analysis, the regulatory region with the detected polymorphisms was classified into three categories: the TATA box including A(TA)7TAA; gtPBREM including c.-3275T>G; and the region with the ten linked polymorphisms specified in the Materials and Methods section above.

**Figure 2:**
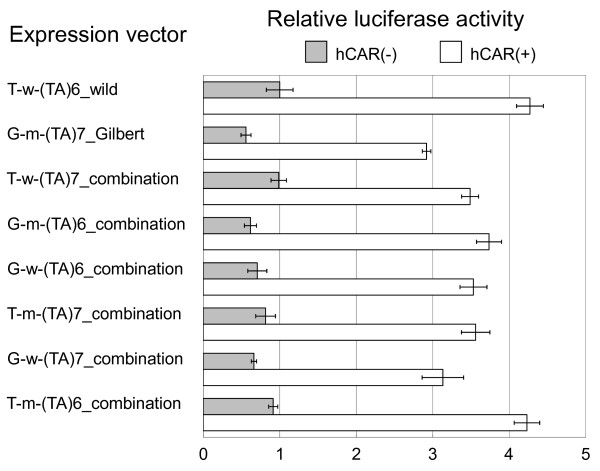
**Transcriptional activity of the continuous 4-kbp regulatory region (c.-4076 to c.-1) with/without the CAR expression plasmid**. The combination of variations in each expression vector is shown in Figure 1. Name of expression vector represents polymorphisms included and vector type. In the first part of the name, T and G respectively denote c.-3275T and c.-3275G. In the second part of the name, w and m refer to wild type and mutant-type region between gtPBREM and the TATA box. In the third part, (TA)6 and (TA)7 denote A(TA)6TAA and A(TA)7TAA. In the fourth part, wild, Gilbert and combination indicate that each vector has wild-type sequence, or typical mutant-type sequence in subjects suffering from Gilbert syndrome, or an artificial sequence constructed for this study. The mean transcriptional activity of the wild-type vector, T-w-(TA)6_wild, without the CAR expression plasmid was chosen as 1.00. Each column and bar represents the mean and SD of four independent determinations. The vector with typical regulatory region showing Gilbert syndrome is G-m-(TA)7_Gilbert. The open box is a wild-type region between gtPBREM and the TATA box, including ten linked polymorphisms: c.-3152G>A, c.-2951A>G, c.-2743T>C, c.-2737T>C, c.-2726G>A, c.-2724AT[8], c.-2473T>G, c.-1352A>C, c.-689A>C and c.-364C>T. The gray box is a mutant-type region having mutant-type sequence corresponding to the wild-type inner region. Luc is luciferase cDNA.

The gray bars in Figure 2 show transcriptional activities without co-expression of CAR. A(TA)7TAA did not significantly reduce transcriptional activity (94% of wild type, *p *= 0.14). In contrast, c.-3275T>G and the ten linked polymorphisms significantly reduced the activity, to 69% and 88% of wild type respectively (*p *< 0.001 and *p *= 0.001). Our study shows that c.-3275T>G exerts the greatest reduction in transcription. The typical haplotype of Gilbert patients (G-m-(TA)7_Gilbert in Figure 2) reduced transcriptional activity to 56% of wild type.

The white bars in Figure 2 show transcriptional activities with co-expression of CAR. The activity of wild-type haplotype was increased with CAR by a factor of 4.27 (S.D. = 0.18, *p *< 0.001). The ten linked polymorphisms alone did not reduce transcriptional activity. In contrast, c.-3275T>G and A(TA)7TAA significantly reduced the activity, to 85% and 83% of wild type respectively (*p *< 0.001 and *p *< 0.001). The typical haplotype of Gilbert syndrome patients (G-m-(TA)7_Gilbert in Figure 2) reduced transcriptional activity to 68% of wild type. These results suggest that AT(TA)7TAA by itself does not reduce transcriptional activity enough to cause Gilbert syndrome, and that co-operation of c.-3275T>G and the ten linked polymorphisms with AT(TA)7TAA is necessary to cause the syndrome.

## Discussion

By reducing the transcription of UGT1A1, A(TA)7TAA is acknowledged to be one of the most important causes of Gilbert syndrome. Further polymorphisms located upstream of the UGT1A1-coding region have recently been detected but, apart from A(TA)7TAA and -3275T>G at gtPBREM, the role of these polymorphisms in the development of Gilbert syndrome is currently unclear.

In the present analysis of the regulatory region of *UGT1A1*, nine haplotypes were detected with differing combinations of 14 polymorphic variations (Table 2). From this analysis we were able to determine the typical haplotype of the regulatory region of patients with A(TA)7TAA as c.-3275G,.c-3152A, c.-2951G, c.-2743C, c.-2737C, c.-2726A, c.-2724AT[8] or [>8], c.-2473G, c.-1352C, c.-1125C, c.-997G, c.-689C, c.-364T and A(TA)7TAA. In addition, the regulatory region of wild type was identical with NG_002601 in all Japanese and Caucasian cases, and different from the sequence used in previous expression studies, namely AF297093 in GenBank [12,28]. Specifically, four base-pairs (c.-3043T, c.-3032G, c.-2985C and c.-2982G) downstream near gtPBREM in AF297093 do not exist in NG_002601.

We classified the regulatory region into three categories so as to simplify the analysis, taking account of previous studies and the locations found in the regulatory sequence. Those three categories are: gtPBREM including c.-3275T>G; the region including ten linked polymorphisms; and the TATA box including A(TA)7TAA. We further examined their effect on transcription, both in the presence and absence of CAR. This was done for three reasons. First, enhancer activity of gtPBREM is elevated by CAR in *in-vitro *experiments using culture cells [10]. Second, CAR has been proposed as a key regulator of bilirubin clearance in the liver in wild type and humanized CAR transgenic mice [29]. Third, c.-3275T>G is reported to be a further factor for the development of hyperbilirubinemia [30].

Transcription of the wild-type regulatory region was enhanced 4.3 times by co-expression of CAR (Figure 2). This was a similar factor to that in previous studies of UGT1A1 [10]. There were differences in transcription between A(TA)7TAA, c.-3275T>G and the ten linked polymorphisms, both in the presence and the absence of CAR. A(TA)7TAA by itself reduced transcription to 94% of wild type (p = 0.14, not significant) without CAR, and to 83% (p < 0.001) with CAR. By itself, then, A(TA)7TAA does not reduce transcriptional activity to 42-50%, which is the value estimated from the enzyme activity and the amount of enzyme protein in human liver samples of homozygous A(TA)7TAA [18-20]. A(TA)7TAA likewise does not reduce transcription down to the values found in human samples in our experiment using shorter regulatory regions (c.-2323 to c.-1 and c.-274 to c.-1) (data not shown). These results suggest that further mutations/variations linked to A(TA)7TAA participate in reducing the transcription.

The ten linked polymorphisms located between A(TA)7TAA and c.-3275T>G together reduced transcription to 88% (p = 0.001) of wild type without CAR. This reduction might be due mainly to c.-1352A>C, since this variation reduced the activity to 85% of wild type in our previous study with a 3-kbp regulatory region [21]. The ten linked polymorphisms did not induce any significant change in transcription with CAR. It is not clear why there was no significant reduction of transcription in the presence of CAR, but the 4.3-fold enhancement of the activity with CAR might mask the inhibitory effect of c.-1352A>C.

Unlike A(TA)7TAA and the ten linked polymorphisms, c.-3275T>G noticeably reduced transcriptional activity both with and without CAR, to 69% (p < 0.001) and 85% (p < 0.001) of wild type respectively. This result is consistent with the report that c.-3275T>G is a factor for the development of hyperbilirubinemia [30].

Sugatani et al. recently reported that the TATA box reduced transcription to a greater extent than c.-3275T>G (c.-3279T>G on their sequence), in a study using the regulatory region from c.-3570 to c.-1 [28]. This regulatory region has two significant differences in their work and ours. First, they did not consider the ten polymorphisms between gtPBREM and the TATA box. Second, their study used the sequence of AF297093 whereas we used NG_002601. At least part of the discrepancy between their results and ours might stem from these differences.

Without CAR, the typical haplotype of Gilbert syndrome patients was effective in reducing transcription, to 56% of wild type, and with CAR to 68% of wild type (Figure 2). The results without CAR are almost compatible with previous *in vivo *data: UGT1A1 enzyme activity was 48%, and the expression level of UGT1A1 protein was 42-50%, of wild type in studies using human liver samples of carriers with homozygous A(TA)7TAA [18-20]. There is only a small amount of CAR in the nuclei of human primary hepatocytes [31], but it has been reported that defects in bilirubin clearance are not observed in CAR-null mice until the appropriate stress is applied, and that nuclear CAR increases in the presence of activators such as a large concentration of bilirubin [29]. Consequently the CAR state of the patients may be similar to those in the experiment without CAR, in which case CAR is not important to serum bilirubin levels of the patient. Even if the amount of nuclear CAR in the patients is intermediate between the values in the experiments with and without CAR, A(TA)7TAA by itself is not enough to reduce transcription to the level found in the liver of patients with homozygous A(TA)7TAA [18-20].

Bilirubin levels are found to be related to polymorphisms of *UGT1A1*; c.-3275T>G, A(TA)7TAA and c.211G>A in epidemiological genetic studies [30,32].

Epidemiological evaluation of the effect of each polymorphism on the development of Gilbert syndrome is difficult, because almost all haplotypes with A(TA)7TAA also have c.-3275T>G and other polymorphisms such as c.-1352A>C.

Our *in-vitro *studies imply the existence of combined effects of the polymorphisms on transcriptional activity in the development of Gilbert syndrome; these studies use vectors with combinations of the polymorphisms designed specifically to analyze the role of the individual polymorphisms. Our results may explain why almost all patients with Gilbert syndrome are double homozygous for A(TA)7TAA and c.-3275T>G [24,33,34]. Since c.-3275T>G alone is not enough to reduce the transcription level to that of the patients, less frequent A(TA)7TAA may play a determinant role in the etiology of Gilbert syndrome. Exceptional patients, such as one who is homozygous for A(TA)7TAA and heterozygous for c.-3275T>G, heterozygous for A(TA)7TAA and homozygous for c.-3275T>G, and homozygous only for c.-3275T>G, may have further variations of *UGT1A1 *that have not yet been investigated, or have variations of the other enzyme(s) related to bilirubin metabolism [28,33,34]. Further studies of the relation between serum bilirubin concentration and genotype that include enough patients with minor haplotypes, based on this analysis, should identify accurately the involvement of each polymorphic mutation in the development of Gilbert syndrome.

## Conclusions

All haplotypes of Gilbert syndrome patients with A(TA)7TAA have both c.-3275T>G and 8-11 polymorphisms between gtPBREM and A(TA)7TAA. CAR increases transcription and changes the amount of inhibition exerted by each polymorphism on transcription. Regardless of the presence of CAR, A(TA)7TAA did not by itself reduce transcriptional activity enough to cause Gilbert syndrome. Our study shows that c.-3275T>G and the ten polymorphisms detected in the patients are also important for the decrease of transcription of *UGT1A1 *in addition to A(TA)7TAA, and that Gilbert syndrome is likely to be caused by the combined effects of these polymorphisms.

## Abbreviations

UGT1A1: bilirubin UDP-glucuronosyltransferase; *UGT1A1*: bilirubin UDP-glucuronosyltransferase gene; gtPBREM: phenobarbital responsive enhancer module of *UGT1A1*; CAR: constitutive androstane receptor; SNP: single nucleotide polymorphism.

## Competing interests

The authors declare that they have no competing interests.

## Authors' contributions

KM took part in the design of the study, performed the experiments and the statistical analysis, and drafted the manuscript. YM and HS took part in the design of the study, coordinated the study and helped to draft the manuscript. YT helped to draft the manuscript. All authors read and approved the final manuscript prior to submission.

## Pre-publication history

The pre-publication history for this paper can be accessed here:

http://www.biomedcentral.com/1471-230X/10/57/prepub

## Supplementary Material

Additional file 1 **Table S1: Results of multiple regression analyses**. Changes in transcriptional activity due to the polymorphisms were analyzed by multiple regression analysis. ACT = A_1_^X1^A_2_^X2^A_3_^X3 ^ACT_0_. Notation: ACT: transcriptional activity; ACT_0_: transcriptional activity of the wild type vector, that is fixed to 1 in our study; A_1_, A_2 _and A_3_: coefficient of c.-3275T>G, of the ten linked polymorphisms and of A(TA)7TAA, respectively; X_1_, X_2 _and X_3_: numbers for dummy coding, defined as 1 in the case of mutant-type polymorphism and 0 in the case of wild-type polymorphism. In the study without CAR, A_1_, A_2 _and A_3 _are 0.693 (*p *< 0.001), 0.876 (*p *= 0.001) and 0.944 (*p *= 0.141) respectively. In the study with CAR, A_1_, A_2 _and A_3 _are 0.854 (*p *< 0.001), 0.997 (*p *= 0.839) and 0.829 (*p *< 0.001) respectively. *P*: *p *value. Click here for file

Additional file 2 **Table S2: Diplotype of Caucasians and Japanese**. We detected 14 kinds of diplotypes in our study from four different groups: Japanese random subjects, Japanese patients with Gilbert syndrome having homozygous A(TA)7TAA, normal Caucasians, and Caucasian patients with Gilbert syndrome. Click here for file
