# Supplementary material for: Combined effect of regulatory polymorphisms on transcription of UGT1A1 as a cause of Gilbert syndrome
Source: BMC Gastroenterol. 2010 Jun 8;10:57. doi: 10.1186/1471-230X-10-57 (PMC2894006; doi:10.1186/1471-230X-10-57)
Supplement: Additional file 1 — Table S1: Results of multiple regression analyses. Changes in transcriptional activity due to the polymorphisms were analyzed by multiple regression analysis. ACT = A1X1A2X2A3X3 ACT0. Notation: ACT: transcriptional activity; ACT0: transcriptional activity of the wild type vector, that is fixed to 1 in our study; A1, A2 and A3: coefficient of c.-3275T>G, of the ten linked polymorphisms and of A(TA)7TAA, respectively; X1, X2 and X3: numbers for dummy coding, defined as 1 in the case of mutant-type polymorphism and 0 in the case of wild-type polymorphism. In the study without CAR, A1, A2 and A3 are 0.693 (p < 0.001), 0.876 (p = 0.001) and 0.944 (p = 0.141) respectively. In the study with CAR, A1, A2 and A3 are 0.854 (p < 0.001), 0.997 (p = 0.839) and 0.829 (p < 0.001) respectively. P: p value. [file 1471-230X-10-57-S1.DOC]

**Table S1: Results of multiple regression analyses (Supplemental Data)**

|  | | | Study 1 | Study 2 |
| --- | --- | --- | --- | --- |
| Co-expression of CAR | | | - | + |
| Independent variable | | |  |  |
| c.-3275T>G | Log_10_A_1_ | RC  SE  SRC  *P* | -0.159  0.016  -0.719  <0.001*** | -0.069  0.007  -0.510  <0.001*** |
|  | A_1_ | | 0.693 | 0.854 |
| Ten linked polymorphisms | Log_10_A_2_ | RC  SE  SRC  *P* | -0.058  0.016  -0.260  0.001** | -0.001  0.007  -0.010  0.839 |
|  | A_2_ | | 0.876 | 0.997 |
| A(TA)7TAA | Log_10_A_3_ | RC  SE  SRC  *P* | -0.025  0.016  -0.112  0.141 | 0.082  0.007  -0.607  <0.001*** |
|  | A_3_ | | 0.944 | 0.829 |
|  | R^2^ | | 0.894 | 0.950 |

Study 1: expression study of the regulatory region (c.-4076 to c.-1) of *UGT1A1* without co-expression of CAR.

Study 2: expression study of the regulatory region (c.-4076 to c.-1) of *UGT1A1* with co-expression of CAR.

Ten linked polymorphisms: c.-3152G>A, c.-2951A>G, c.-2743T>C, c.-2737T>C, c.-2726G>A, c.-2724AT[8], c.-2473T>G, c.-1352A>C, c.-689A>C and c.-364C>T; RC: regression coefficient; SE: standard error; SRC: standardized regression coefficient; *P*: *p* value **p*<0.05, ***p*<0.01, ****p*<0.001

In all analyses, transcriptional activity of the wild type vector was fixed to 1.

Polymorphisms change the transcriptional activity multiplicatively.

Transcriptional activity is therefore based on the formula:

ACT= A_1_^X1^A_2_^X2^A_3_^X3^ ACT_0_ …(1)

ACT: transcriptional activity

ACT_0_: transcriptional activity of the wild type vector that is fixed to 1 in this study

A_1_, A_2_ and A_3_: coefficient of c.-3275T>G, ten linked polymorphisms and A(TA)7TAA, respectively.

X_1_, X_2_ and X_3_: numbers for dummy coding, defined as 1 in the case of mutant-type polymorphism and as 0 in the case of wild-type polymorphism.

For multiple regression analyses, formula (1) is converted logarithmically to the following formula:

Log_10_ACT= X_1_Log_10_ A_1_+X_2_Log_10_ A_2_+X_3_Log_10_ A_3_ + Log_10_ ACT_0_ …(2)

Since Log_10_ ACT_0_ is 0, formula (2) simplifies to the following formula:

Log_10_ACT= X_1_Log_10_ A_1_+X_2_Log_10_ A_2_+X_3_Log_10_ A_3_ …(3)

Multiple regression analyses are based on formula (3).
