# Supplementary material for: Combined effect of regulatory polymorphisms on transcription of UGT1A1 as a cause of Gilbert syndrome
Source: BMC Gastroenterol. 2010 Jun 8;10:57. doi: 10.1186/1471-230X-10-57 (PMC2894006; doi:10.1186/1471-230X-10-57)
Supplement: Additional file 2 — Table S2: Diplotype of Caucasians and Japanese. We detected 14 kinds of diplotypes in our study from four different groups: Japanese random subjects, Japanese patients with Gilbert syndrome having homozygous A(TA)7TAA, normal Caucasians, and Caucasian patients with Gilbert syndrome. [file 1471-230X-10-57-S2.DOC]

**Table S2: Diplotype of Caucasians and Japanese (Supplemental Data)**

| diplotype | Number of subjects | | | |  | gtPBREM |  | Polymorphisms in the region between gtPBREM and TATA box | | | | | | | | | | | |  | TATA box |
| --- | --- | --- | --- | --- | --- | --- | --- | --- | --- | --- | --- | --- | --- | --- | --- | --- | --- | --- | --- | --- | --- |
|  | JRS^a^ | JG7^b^ | NC^c^ | CG^d^ |  | c.-3275 |  | c.-3152 | c.-2951 | c.-2743 | c.-2737 | c. -2726 | c.-2724AT[n] | c.-2473T | c.-1352 | c.-1125 | c.-997 | c.-689 | c.-364 |  | A(TA)nTAA |
| I, I | 20 |  | 3 |  |  | T/T |  | G/G | A/A | T/T | T/T | G/G | 3/3 | T/T | A/A | C/C | G/G | A/A | C/C |  | 6/6 |
| I, II | 16 |  | 2 |  |  | T/**G** |  | G/G | A/A | T/T | T/T | G/**A** | 3/**>8** | T/**G** | A/**C** | C/C | G/**A** | A/**C** | C/C |  | 6/6 |
| I, III |  |  | 1 |  |  | T/T |  | G/G | A/A | T/T | T/T | G/G | 3/3 | T/**G** | A/**C** | C/C | G/**A** | A/**C** | C/C |  | 6/6 |
| I, IV | 2 |  |  |  |  | T/T |  | G/G | A/A | T/T | T/T | G/G | 3/3 | T/T | A/**C** | C/C | G/**A** | A/A | C/C |  | 6/6 |
| I, V | 10 |  |  |  |  | T/**G** |  | G/**A** | A/**G** | T/**C** | T/**C** | G/**A** | 3/**8** | T/**G** | A/**C** | C/C | G/G | A/C | C/**T** |  | 6/**7** |
| I, VI |  |  | 1 |  |  | T/**G** |  | G/**A** | A/**G** | T/**C** | T/**C** | G/**A** | 3/**>8** | T/**G** | A/**C** | C/C | G/G | A/C | C/**T** |  | 6/**7** |
| I, VII |  |  | 1 |  |  | T/**G** |  | G/G | A/**G** | T/**C** | T/T | G/**A** | 3/**>8** | T/**G** | A/**C** | C/C | G/G | A/**C** | C/**T** |  | 6/**7** |
| II, II | 1 |  |  |  |  | **G/G** |  | G/G | A/A | T/T | T/T | **C/C** | **>8/>8** | **G/G** | **C/C** | C/C | **C/C** | **C/C** | C/C |  | 6/6 |
| II, VI | 1 |  | 2 |  |  | **G/G** |  | G/**A** | A/**G** | T/**C** | T/**C** | **A/A** | **>8/>8** | **G/G** | **C/C** | C/C | G/**A** | **C/C** | C/**T** |  | 6/**7** |
| V, V |  | 4 |  |  |  | **G/G** |  | **A/A** | **G/G** | **C/C** | **C/C** | **A/A** | **8/8** | **G/G** | **C/C** | C/C | G/G | **C/C** | **T/T** |  | **7/7** |
| VI, VI |  |  |  | 5 |  | **G/G** |  | **A/A** | **G/G** | **C/C** | **C/C** | **A/A** | **>8/>8** | **G/G** | **C/C** | C/C | G/G | **C/C** | **T/T** |  | **7/7** |
| VI, VIII |  |  |  | 1 |  | **G/G** |  | G**/A** | **G/G** | **C/C** | **C/C** | **A/A** | **>8/>8** | **G/G** | **C/C** | C/**T** | G/G | **C/C** | **T/T** |  | **7/7** |
| VII, VII |  |  |  | 2 |  | **G/G** |  | G/G | **G/G** | **C/C** | T/T | **A/A** | **>8/>8** | **G/G** | **C/C** | C/C | G/G | **C/C** | **T/T** |  | **7/7** |
| VII, VIII |  |  |  | 3 |  | **G/G** |  | G/G | **G/G** | **C/C** | T/**C** | **A/A** | **>8/>8** | **G/G** | **C/C** | C/**T** | G/G | **C/C** | **T/T** |  | **7/7** |

Boldfaces indicate variations.

^a^JRS, Japanese random subjects; ^b^JG7, Japanese patients with Gilbert syndrome having homozygous A(TA)7TAA; ^c^NC, normal Caucasians; ^d^CG, Caucasian patients with Gilbert syndrome; ^e^Numbers in parentheses indicate the number of total alleles in each group
